# Oxidative Metabolism of Rye (*Secale cereale* L.) after Short Term Exposure to Aluminum: Uncovering the Glutathione–Ascorbate Redox Network

**DOI:** 10.3389/fpls.2016.00685

**Published:** 2016-05-24

**Authors:** Alexandra de Sousa, Hamada AbdElgawad, Asard Han, Jorge Teixeira, Manuela Matos, Fernanda Fidalgo

**Affiliations:** ^1^Biosystems and Integrative Sciences Institute, Departamento de Biologia, Faculdade de Ciências, Universidade do PortoPorto, Portugal; ^2^Laboratory for Integrated Molecular Plant Physiology Research, Department of Biology, University of AntwerpAntwerp, Belgium; ^3^Departamento de Genética e Biotecnologia, Universidade de Trás-os-Montes e Alto-DouroVila Real, Portugal; ^4^Biosystems and Integrative Sciences Institute, Faculdade de Ciências, Universidade de LisboaLisboa, Portugal

**Keywords:** aluminum, *Secale cereale* L., oxidative metabolism, ascorbate–glutathione cycle, Al short-term exposure, antioxidant response

## Abstract

One of the major limitations to plant growth and yield in acidic soils is the prevalence of soluble aluminum ions (Al^3+^) in the soil solution, which can irreversible damage the root apex cells. Nonetheless, many Al-tolerant species overcome Al toxicity and are well-adapted to acidic soils, being able to complete their life cycle under such stressful conditions. At this point, the complex physiological and biochemical processes inherent to Al tolerance remain unclear, especially in what concerns the behavior of antioxidant enzymes and stress indicators at early plant development. Since rye (*Secale cereale* L.), is considered the most Al-tolerant cereal, in this study we resort to seedlings of two genotypes with different Al sensitivities in order to evaluate their oxidative metabolism after short term Al exposure. Al-induced toxicity and antioxidant responses were dependent on rye genotype, organ and exposure period. Al affected biomass production and membrane integrity in roots and leaves of the sensitive (RioDeva) genotype. Catalase was the primary enzyme involved in H_2_O_2_ detoxification in the tolerant (Beira) genotype, while in RioDeva this task was mainly performed by GPX and POX. Evaluation of the enzymatic and non-enzymatic components of the ascorbate–glutathione cycle, as well the oxalate content, revealed that Beira genotype coped with Al stress by converting DHA into oxalate and tartarate, which posteriorly may bind to Al forming non-toxic chelates. In contrast, RioDeva genotype used a much more ineffective strategy which passed through ascorbate regeneration. So, remarkable differences between MDHAR and DHAR activities appear to be the key for a higher Al tolerance.

## Highlight

At early development stages the underling physiological and biochemical mechanisms to tolerate Al passes through modulation of ascorbate–glutathione enzymes, essentially MDHAR and DHAR in a genotype-dependent manner.

## Introduction

Approximately 30–40% of the world’s potentially arable lands are acidic soils, on which Al is the main factor that limits plant growth and crop production (Kochian et al., 2004). Soils acidification worldwide results from natural and anthropogenic inputs. When soil pH is below 5.0, the phytotoxic ion Al^3+^ is released into the soil solution resulting in both impaired root growth and early seedling development (Sasaki et al., 2002). Diverse management strategies were used by seed companies and farmers’ associations over the last decades to increase soil pH and reduce Al toxicity. Direct application of lime was the most common method employed. However, disadvantages inherited to this method prompted producers to seek a viable and sustainable solution for this problem (Cassiolato et al., 2000). Observing that some plant species exhibited natural tolerance to Al toxicity in fields, producers started to ask for the development of new crop varieties with similar characteristics that would allow them to direct future agricultural expansion onto acid soils. Since a great inter- and intraspecific variability has been observed for Al tolerance, several efforts are in progress in order to identify major genes and associated biochemical and physiological processes underlying Al resistance in Al-tolerant genotypes. This will provide important resources for further improvement of crop resistance for Al toxicity trough breeding programs. Thus, it is crucial to thoroughly exploit tolerance mechanisms that operate in early stages of seedling development of Al-tolerant genotypes that lately are able to complete their life cycle under Al stress conditions. Rye (*Secale cereale*) is considered the most Al-tolerant species among Triticeae, but research concerning rye’s Al tolerance is extremely limited (Aniol and Gustafson, 1984; Ryan et al., 2011). Uncovering the genetic, molecular and physiological mechanisms involved in rye Al tolerance will provide vital information that can be used to increase Al tolerance in other cereals, such as wheat and Triticale.

Several mechanisms involved in the external and/ or internal Al tolerance have been proposed (Huang et al., 2009; Brunner and Sperisen, 2013). However, root exudation of organic acids under Al stress seems to play a central role in Al detoxification in several plant species or cultivars, since they can form stable, non-toxic complexes with Al^3+^ at the rizhosphere. In fact, several genes controlling this trait were identified among cereals, including rye (Ma et al., 2001; Hede et al., 2002; Fontecha et al., 2007; Yokosho et al., 2010; Ryan et al., 2011). Organic acids, namely oxalic acid have also been implicated in internal Al^3+^ tolerance once it has entered into root and shoot symplast (Brunner and Sperisen, 2013). Al detoxification is achieved by complexation with oxalate in a 1:1, 1:2, or 1:3 molar ratios, followed by sequestration into vacuoles (Ma, 2000). It’s a well-known fact that plant exposure to environmental stresses, including metal toxicity, leads to the formation of peroxides and free radicals that cause damage to proteins, lipids and carbohydrates (Sharma et al., 2012). There has been increasing evidence that Al affects ROS homeostasis in several plant species. Al triggered ROS production in potato (Tabaldi et al., 2009), rice (Kuo and Kao, 2003), soybean (Cakmak and Horst, 1991), and tobacco (Yamamoto et al., 2002) leading to membrane lipid peroxidation (LP) and consequently, to highly toxic lipid peroxy radicals (Yin et al., 2010). Cell membranes are major targets of Al toxicity in many Al-sensitive species. Al binds to phospholipids and proteins of the plasma membrane altering membrane permeability, fluidity and electrochemical potential. Also, Al interacts with cation channels indirectly suppressing H^+^-ATPase activity (Zhang and Taylor, 1991; Matsumoto, 2000; Horst et al., 2010). In maize, rather than LP, Al stress resulted in protein oxidation (Boscolo et al., 2003). Therefore, Al-induced inhibition of root growth may be related to oxidative stress and changes in cell wall biomechanical properties (Yamamoto et al., 2003). To cope with additional ROS-generating mechanisms, plants developed an efficient enzymatic and non-enzymatic antioxidant defense system which controls the cascades of oxidation and protects plant cells from oxidative damage by scavenging ROS (Sharma et al., 2012). Superoxide dismutase (SOD) is a key antioxidant enzyme considered to be the first line of defense against oxidative stress, since it catalyzes the dismutation of superoxide anion (O_2_^∙-^) into H_2_O_2_ and oxygen (O_2_). H_2_O_2_ is then reduced to water (H_2_O) by CAT, APX, GPX, and other POX (Sharma et al., 2012). Ascorbate, carotenoids, flavonoids, gluthatione, phenols, proline, and tocopherols represent the non-enzymatic metabolites of the antioxidant defense system (Sharma et al., 2012). Ascorbate, gluthatione, and proline are powerful antioxidants since they can directly scavenge reactive oxygen species (ROS) like singlet oxygen (_1_O^2^) and hydroxyl radicals (HO^∙-^; Foyer and Noctor, 2011; Xu et al., 2012). Ascorbate and gluthatione also serve as electron donors for key enzymes such as APX and GPX and are involved in several physiological mechanisms like cell division and expansion (Noctor et al., 2012). Besides APX, other enzymes involved in the ascorbate–glutathione cycle (ASC-GLU, MDHAR, DHAR, GR) also play an important role in oxidative stress management (Sharma et al., 2012).

However, until now it remains unclear how Al affects cellular redox homeostasis of rye seedlings after short-term exposure and what molecular mechanisms differ between tolerant and sensitive genotypes. In this work analyses were performed that unravel the physiological and biochemical basis of Al toxicity and tolerance in rye genotypes with emphasis on the antioxidant metabolism. The results allow us to understand how rye genotypes are capable of modulating their metabolic and physiological responses in order to cope with Al stress in early development stages. We also observed that metabolic modifications are organ specific within each genotype, and how it reflected on different morphological characteristics.

## Materials and Methods

### Rye Lines and Experimental Setup

Seeds of two rye genotypes, an Al-tolerant cultivar, Beira (Portuguese Regional Cultivar), and an Al-sensitive, RioDeva (Spanish inbreed line), were kindly provided by University of Trás-os-Montes e Alto Douro (UTAD/Vila Real/Portugal). Surface-sterilized seeds [sodium hypochlorite 5% (w/v), 10 min] were rinsed in sterile deionized water and germinated in the dark at 25°C in Petri dishes. Seedlings were hydroponically cultured in a modified Hoagland’s solution containing: CaCl_2_ 22.20 g L^-1^, KNO_3_ 32.86 g L^-1^, MgCl_2_.6H_2_O 25.41 g L^-1^, (NH_4_)_2_SO_4_ 0.66 g L^-1^, NH_4_NO_3_ 1.60 g L^-1^ and maintained at 25°C under a 16/8 h photoperiod with a photosynthetically active radiation (PAR) of 60 μmol m^-2^ s^-1^. After 48 h roots and leaves were collected (0 h; 0 mg L^-1^) and the remaining seedlings were transferred to a new nutritive solution with the same mineral composition described above, supplemented with 5 mg L^-1^ of Al^3+^ in the form of AlCl_3_^.^6H_2_O. Samples of roots and leaves were collected 24 and 48 h after exposure to the Al treatment. Again, the remaining seedlings were transferred to a new nutritive solution without Al (recovery treatment) and samples were collected 48 h later (96 h; 0 mg L^-1^). Biomass production of rye seedlings was determined as fresh weight for each experimental condition. Root length was determined after the recovery period. The nutrient solution was continuously aerated and the pH was maintained at 4.0 throughout the assays. All samples were immediately frozen in liquid nitrogen and grinded to a fine powder and finally stored at -80°C for biochemical analyzes. Negative controls of selected parameters were performed in a second group of plants grown on the same conditions stated before, but without Al in order to differentiate between Al toxicity effects and development effects.

### Al Tolerance Screening Tests

The Al tolerance screening test was performed by a modified-pulse method (Aniol and Gustafson, 1984). After the recovery treatment, seedlings were washed 3 min with running distilled water and stained with 0.1% (w/v) *Eriochrome cyanine R* for 10 min. This dye forms a stable bluish complex with Al. Excess dye was washed from rye roots and seedlings were transferred to Al- free nutrient solution for 48 h. Al tolerance was measured as root regrowth of seedlings.

### Oxidative Stress Parameters

Lipid peroxidation was determined according to Murshed et al. (2008). Briefly, 100 mg of frozen tissue were homogenized (MagNALyser, Roche, Vilvoorde, Belgium; 1 min, 7000 rpm) in 1 mL of 80% (v/v) ethanol. After centrifugation (12, 000 *g*, 15 min) 0.5 mL of the supernatant was added to 1 mL of 0.5% (w/v) TBA in 20% (w/v) TCA. The samples were incubated at 95°C for 30 min, and the reaction stopped by placing the reaction tubes in an ice bath (10 min) followed by a centrifugation for 2 min at 10, 000 *g*. Absorbance was measured at 450, 532, and 600 nm in a microplate reader. TBARS equivalents were calculated by the following formula: [6.45 x (Abs_532_-Abs_600_) - 0.56 x Abs_450_] and expressed as nmol MDA g^-1^ FW. EL was measured according to the method of Lutts et al. (1996). Seedlings were washed several times with deionized water and, after drying, tissue samples were immersed in 10 mL of deionized water and incubated at 25°C in a rotary shaker (100 rpm). Electrical conductivity of the bathing solution (*T1*) was recorded after 24 h. Samples were immediately autoclaved at 120°C for 20 min and a last conductivity reading (*T2*) was obtained when the solutions reached 25°C. EL was expressed following the formula: [I (%) = [1-(1-T_1_-T_2_) / (1-(C_1_-C_2_))] x 100], where C corresponds to the control situations and T to the treated samples. An Amplex Red Hydrogen Peroxide/Peroxidase Assay Kit (Molecular Probes, Eugene, OR, USA) was used to measure H_2_O_2_ production.

### Scavenging Activity of Hydrogen Peroxide (H_2_O_2_)

Scavenging activity of H_2_O_2_ was determined according to Ngonda (2013). Samples were washed with distilled water and dried at 55°C during 48 h. After, leaf and root tissues (500 mg) were grinded into a fine powder and mixed with 2 mL of 95% (v/v) MeOH. The residue was re-extracted under the same conditions until the supernatant became achromatic. Excess MeOH was removed from extracts in a rotary evaporator at 40°C. Dry extracts were stored at -20°C until further analysis. Briefly, a solution of H_2_O_2_ was prepared in phosphate buffer (pH 7.4). Plant extracts (100 μg/mL) were added to 0.6 mL of H_2_O_2_ solution and the final volume of 3 mL was made by adding the phosphate buffer (pH 7.4). The absorbance of the reaction mixture was measured at 230 nm against a blank solution. The percentage of H_2_O_2_ scavenging by the rye extracts were calculated as:

$$\%\;\mathrm{Scavenged}\;[H_{2}O_{2}]\;=\;[(A_{0}\;-\;A_{1}){/A}_{0}]\times 100$$

Where, A_0_ - Absorbance of control;

A_1_ - Absorbance of extracts.

### Non-enzymatic Antioxidants

Ascorbate and glutathione levels were determined by HPLC analyses (Potters et al., 2004). Samples were extracted in 1 mL of ice-cold 6% (w/v) meta-phosphoric acid and after centrifugation (16, 000 *g*, 4°C, 10 min) antioxidants were separated on a reverse-phase column (100 mm × 4.6 mm Polaris C18-A, 3 μm particle size; 40°C, Varian, CA, USA) with an isocratic flow rate of 1 mL min^-1^ of the elution buffer: 2 mM KCl, pH 2.5 adjusted with *O*-phosphoric acid. Antioxidants were quantified using an electrochemical detector and the purity and identity of the peaks were confirmed using an in-line DAD (SPD-M10AVP, Shimadzu). Total antioxidant concentration was determined after reduction of samples with 0.04 M DTT, for 10 min in obscurity and the redox status was calculated as the ratio of the reduced form to the total antioxidant concentration. Tocopherols (α, β, γ, δ) were determined by HPLC analysis according to AbdElgawad et al. (2015). Tocopherols were extracted with hexane using the MagNALyser (Roche, Vilvoorde, Belgium; 1 min, 7000 rpm). The dried extract (CentriVap concentrator, Labconco, KS, USA) was resuspended in hexane, and tocopherols were separated and quantified by HPLC (Shimadzu, ‘s-Hertogenbosch, The Netherlands; normal phase conditions, Particil Pac 5 μm column material, length 250 mm, i.d. 4.6 mm). Dimethyl tocol (DMT) was used as internal standard (5 ppm). Data were analyzed with Shimadzu Class VP 6.14 software. The TAC of plant extracts was determined by a modified ferric ion reducing antioxidant power (FRAP) assay (Benzie and Szeto, 1999). Antioxidants were extracted by grinding 100 mg of frozen plant tissue in 1 mL of 80% (v/v) ethanol. After centrifugation (3, 000 *g*, 4°C, 15 min) the FRAP reagent (0.3 M acetate buffer, pH 3.6, 0.01 mM TPTZ in 0.04 mM HCl, 0.02 M FeCl_3_.6H_2_O) was mixed with the extracts and measured at 600 nm, using Trolox as a standard.

### Enzyme Assays

Lipoxygenase (EC 1.13.11.12) was extracted in 50 mM sodium phosphate buffer (pH 7.0) containing 1 mM EDTA, 0.1 mM PMSF, 2% (w/v) PVP, 1% glycerol and 0.1% Tween 20. After centrifugation (15, 000 *g*, 4°C, 20 min), 2.9 mL of the assay solution (1 mM linoleic acid in 0.1 M sodium acetate buffer) was added to 0.1 mL of the plant extract and absorbance was measured at 240 nm. LOX activity was calculated using the extinction coefficient of conjugated dienes (𝜀_340_ = 25 mM^-1^ cm^-1^; Ramakrishna and Rao, 2012).

Catalase (EC 1.11.1.6) and GPX (EC 1.11.1.7) were extracted in a 50 mM potassium phosphate (pH 7.0) containing 0.4 mM EDTA, 0.2 mM PMSF, 2% (w/v) insoluble PVPP and 1 mM ascorbic acid. Other antioxidant enzymes such, APX (EC 1.11.1.11), MDHAR (EC 1.6.5.4), DHAR (EC 1.8.5.1), GR (EC 1.8.1.7), and POX (EC 1.11.1.7) were extracted in 50 mM MES/KOH buffer (pH 6.0), containing 2 mM CaCl_2_, 40 mM KCl, and 1 mM ascorbic acid. CAT activity was determined spectrophotometrically at 240 nm by monitoring the rate of H_2_O_2_ decomposition at pH 7.0 (Aebi, 1984). APX, MDHAR, DHAR, and GR activities were determined according to Murshed et al. (2008). GPX activity was calculated by measuring the decrease in NADPH absorbance at 340 nm (Drotar et al., 1985). Peroxidase activity was determined by the oxidation of pyrogallol (𝜀_340_ = 2.47 mM^-1^ cm^-1^; Kumar and Khan, 1982). The soluble protein content was obtained (Lowry et al., 1951) and activity measurements were scaled down for semi-high throughput measurement using a micro-plate reader (Synergy Mx, Biotech Instruments, Inc., Winooski, VT, USA).

### Amino Acids Measurements

Ethanolic extracts of rye seedlings were used to assay free amino acids (FAAs) levels using a Waters Acquity UPLC-tqd system (Milford, MA, USA) equipped with a BEH amide 2.1 × 50 column according to Sinha et al. (2013) with the minor modification previously described by AbdElgawad et al. (2015).

### Oxalate Quantification

Plant samples (100 mg FW) were homogenized in phosphoric acid (0.1%; containing 0.003% butylated hydroxyanisole) using a MagNALyser. The extract was centrifuged at 14, 000 *g* for 30 min at 4°C. The supernatants were passed through Millipore micro filters (0.2 μM pore size). Oxalate was detected by HPLC using a SUPELCOGEL C-610H column (300 mm × 7.8 mm, Supelco, Sigma, St. Louis, MO, USA) coupled to UV detection system set at 210 nm (LaChrom L-7455 diode array, LaChrom, Tokyo, Japan). The mobile phase was a 0.1% phosphoric acid at a flow rate of 0.45 mL min^-1^. Organic acids were quantified using a calibration curve obtained with the corresponding standards.

### Statistical Analysis

Results were expressed as mean ± SD (standard deviation) and analyzed by two-way ANOVA using IBM SPSS Statistica 23 software package (SPSS^®^ Inc., Chicago, IL, USA) for windows, with organs and concentrations used as fixed variables. Data were tested for normal distribution and homogeneity and normalized when necessary. The significance level of 0.05 was used for rejection of the null hypothesis. In cases of significant interactions between factors, one-way ANOVA analysis was performed for each factor, and Tukey’s multiple range tests were used for determining significant differences among means. All experiments were carried out in quadruplicate (*n* = 4), except for biomass determination, where *n* = 30, and root length, where *n* = 100.

## Results

### Plant Growth Response

**FIGURE 1:**
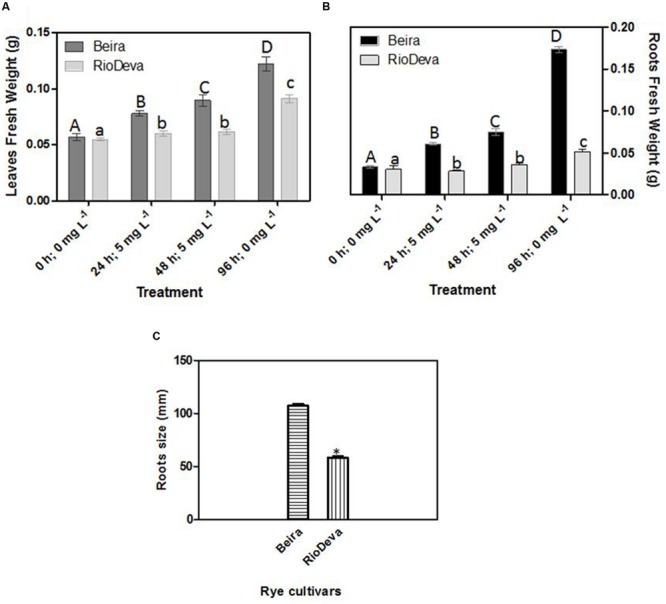
**Biometric analysis: fresh weight in leaves **(A)** and roots **(B)** of rye Al-tolerant (Beira) and Al-sensitive (RioDeva) genotypes and measurement of root length **(C)** after the recovery period (96 h; 0 ppm).** Different uppercase letters represent significant differences between times in Beira, while lowercase letters represent significant differences between times in RioDeva. Values represent mean ± SD (*n* = 30). Asterisk represents significant differences between genotypes in root length. Values represent mean ± SD (*n* = 100).

Significant differences in growth have been observed between rye genotypes, with higher biomass in Beira seedlings for both leaves and roots (**Figures 1A,B**). In RioDeva leaves no significant increase in fresh weight was observed between the beginning (24 h; 5 mg L^-1^) and end of the Al treatment (48 h; 5 mg L^-1^). During recovery period, leaf biomass increased by 44 and 80% in Beira and RioDeva seedlings, respectively, compared to seedlings exposed 48 h to Al treatment (**Figure 1A**). Despite RioDeva roots presented the same response as leaves, Al did not significantly affected root development in Beira seedlings (**Figure 1B**). Biomass of RioDeva roots also increased by 25% during recovery period (96 h, 0 mg L^-1^). Measurements carried after the recovery period demonstrated that RioDeva root length was 45% inferior to the tolerant genotype (**Figure 1C**). Leaves, as well as roots, of Beira and RioDeva seedlings grown in the absence of Al exhibited similar biomass at different time points. This indicates that Al, rather than developmental processes, is responsible for the observed decline in biomass, especially in the sensitive RioDeva genotype (Supplementary Figure S1).

### Al Screening Tolerance

**FIGURE 2:**
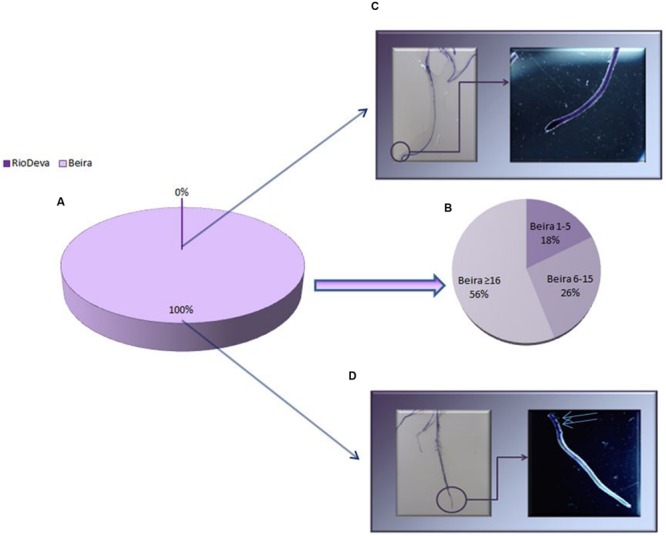
**Root regrowth based on *Eriochrome cyanine R* coloration method for Al toxicity assay.** Mean lengths of root regrowth presented as percentage (%) in both genotypes **(A)**, with Al-tolerant genotype divided in three different classes (1–5 mm, 6–15 mm and ≥ 16 mm; **B**). Al accumulation in roots of RioDeva **(C)** and Beira **(D)** cultivars observed under a stereomicroscope. Scale bars in panels equal 1 mm. Blue arrows represent lateral roots with Al accumulation (*n* = 100).

Al tolerance varies among rye genotypes and is often based on root regrowth after short periods of Al exposure. After the recovery treatment all the seedlings of Beira presented root regrowth, while roots of the Al-sensitive seedlings did not presented any regrowth (**Figures 2A,C,D**). It was also noticed that 56% of root regrowth in Beira seedlings was equal or superior to 16 mm (**Figure 2B**). Optical microscopic observations of roots (data not shown) demonstrated that after the recovery period, Beira seedlings accumulated Al in large amounts in lateral root primordia (LRP) and in the hair roots (HRs). Al accumulation in RioDeva genotype was observed in all cells of root tips and in the vascular tissues. HR and LRP were less abundant in this cultivar and did not present any signals of Al accumulation.

### Oxidative Stress – MDA Content, Electrolyte Leakage, LOX Activity, and H_2_O_2_ Content

**FIGURE 3:**
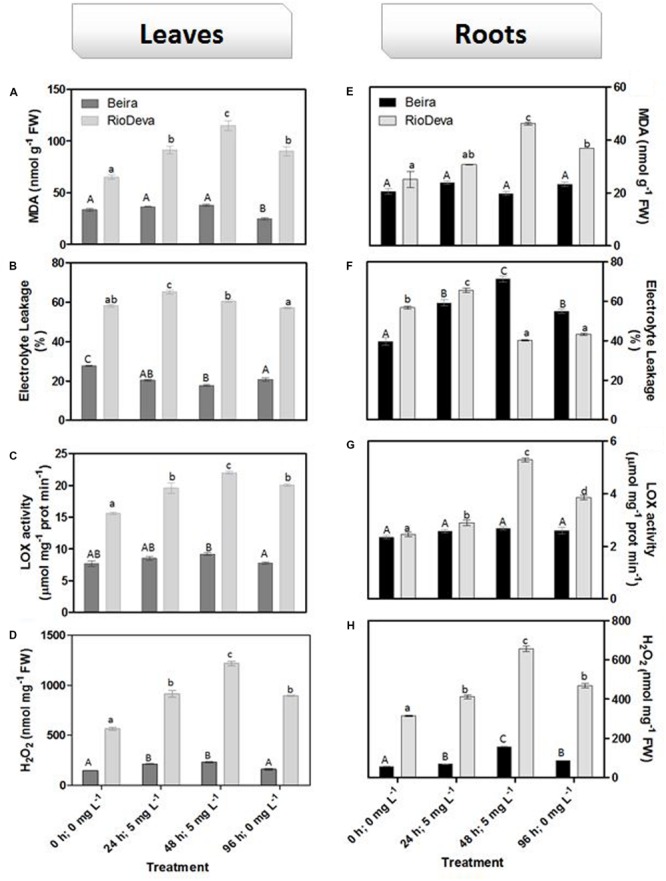
**Oxidative stress parameters of rye Al-tolerant (Beira) and Al-sensitive (RioDeva) genotypes.** Lipid peroxidation (MDA) content **(A,E)**, EL **(B,F)**, LOX activity **(C,G)** and H_2_O_2_ levels **(D,H)** in leaves and roots of rye seedlings, respectively. Different uppercase letters represent significant differences between times in Beira, while lowercase letters represent significant differences between times in RioDeva. Values represent mean ± SD (*n* = 4).

Oxidative membrane damage was more severe in the sensitive genotype under all experimental conditions. The highest values of MDA were found in leaves of the sensitive RioDeva genotype 48 h after exposure to Al, increasing 78% when compared to control seedlings. In the recovery period (96 h; 0 mg L^-1^), MDA content decreased significantly almost reaching the values quantified in the control situation (0 h, 0 mg L^-1^; **Figure 3A**). Roots of RioDeva seedlings presented a similar behavior to those found in leaves, while no fluctuations in MDA levels were found in roots of Beira seedlings (**Figure 3D**). Changes in EL and LOX activity followed the same tendencies as MDA accumulation, except in roots of both genotypes at the end of Al treatment (48 h; 5 mg L^-1^) and in the recovery period (96 h; 0 mg L^-1^; **Figures 3B,C,E–G**). H_2_O_2_ levels were higher in leaves of both genotypes when compared to roots; however, both organs of the Al-sensitive genotype, presented the highest concentration of this ROS (**Figures 3D,H**). Late exposure of rye seedlings to Al (48 h, 5 mg L^-1^) resulted in a 1.5- and 2.2-fold increase in H_2_O_2_ content in leaves of Beira and RioDeva genotypes, respectively. A significant decrease was also noticed in the recovery period (**Figure 3D**). Changes in H_2_O_2_ levels in roots followed the same tendency as leaves for both genotypes (**Figure 3H**). Seedlings grown in the absence of Al showed that differences in the oxidative parameters were majorly due to the effect of Al treatment, in both organs of the rye Al-tolerant and Al-sensitive genotypes (Supplementary Figure S2).

### ROS Homeostasis – H_2_O_2_ Scavenging Activity

**FIGURE 4:**
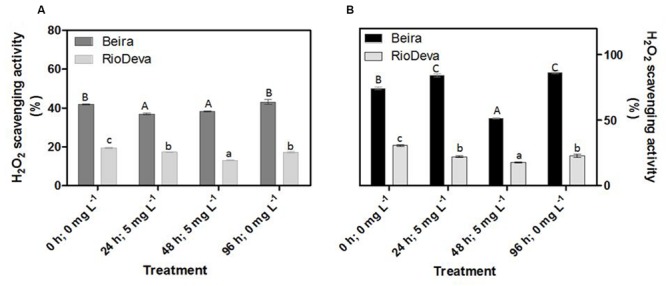
**Hydrogen peroxide scavenging activity of the methanolic extracts of rye Al-tolerant (Beira) and Al-sensitive (RioDeva) genotypes.** Panels represent, H_2_O_2_ scavenging activity **(A,B)** the in leaves and roots, respectively. Different uppercase letters represent significant differences between times in Beira, while lowercase letters represent significant differences between times in RioDeva. Values represent mean ± SD (*n* = 4).

Compared to the sensitive genotype, H_2_O_2_ scavenging activity in leaves was higher in Beira genotype; decreasing 12% after early exposure to Al. In RioDeva leaves H_2_O_2_ scavenging activity decreased 11 and 33% after 24 and 48 h after Al treatment (**Figure 4A**). In roots, H_2_O_2_ scavenging activity was higher in Beira seedlings, decreasing 31% after 48 h of Al exposure. For the same period, H_2_O_2_ scavenging activity in RioDeva roots decreased 42% (**Figure 4B**).

### Cellular Redox Homeostasis – Enzymatic and Non-enzymatic Response

**FIGURE 5:**
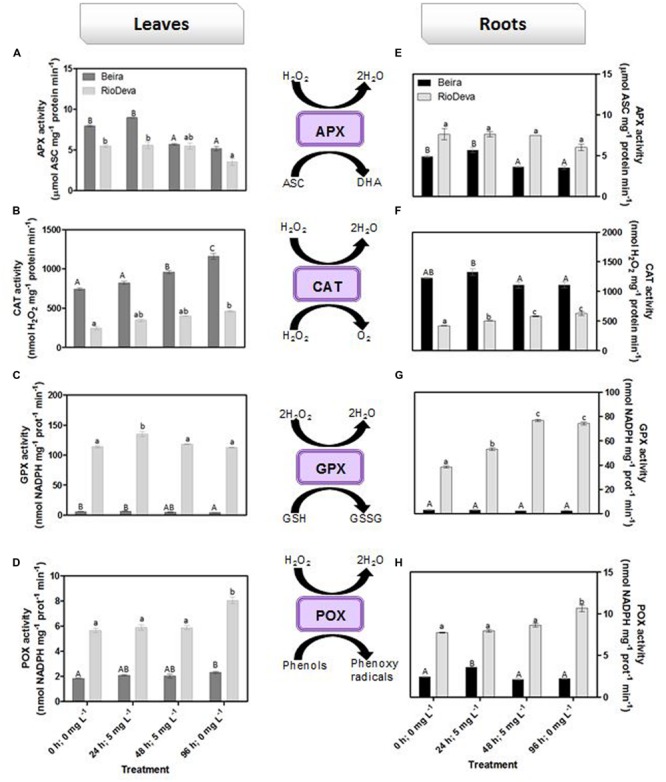
**Response of ROS scavenging enzymes in rye Al-tolerant (Beira) and Al-sensitive (RioDeva).** Panels represent APX activity **(A,E)**, catalase activity **(B,F)**, GPX activity **(C,G)** and POX activity **(D,H)**, in leaves and roots of rye seedlings, respectively. Different uppercase letters represent significant differences between times in Beira, while lowercase letters represent significant differences between times in RioDeva. Values represent mean ± SD (*n* = 4).

**FIGURE 6:**
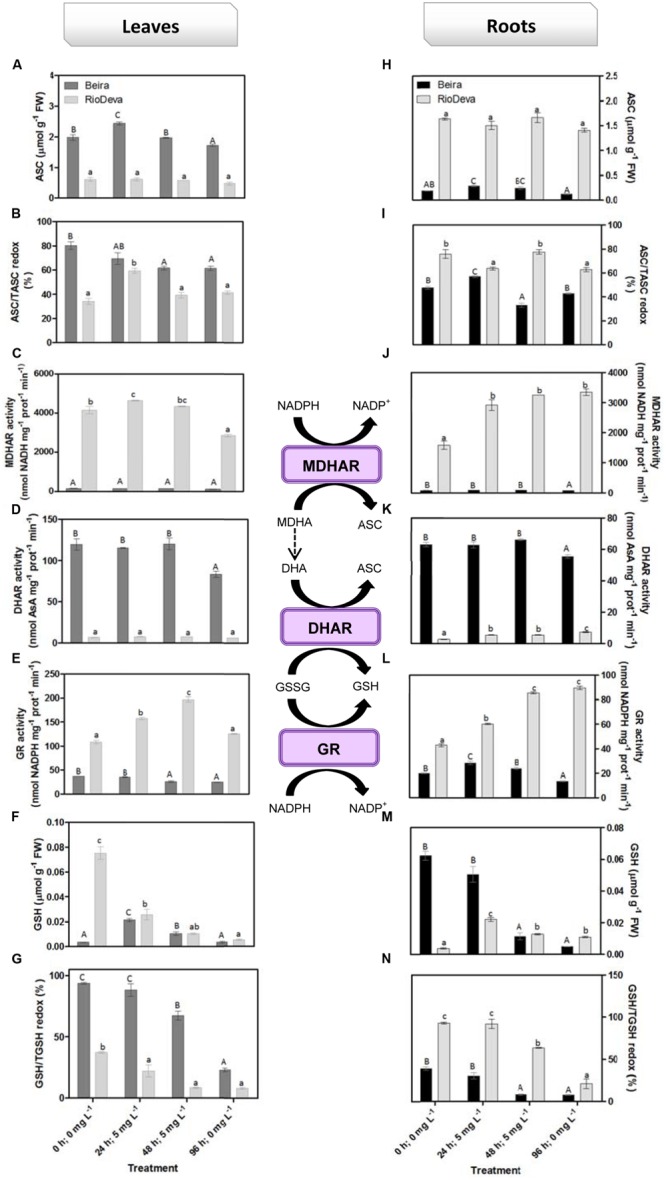
**Response of the ascorbate–glutathione defense in rye Al-tolerant (Beira) and Al-sensitive (RioDeva) genotype.** Reduced ascorbate levels **(A,H)**, ascorbate redox status (ASC/tASC; **B,I**), MDHAR activity **(C,J)**, DHAR activity **(D,K)**, GR activity **(E,L)**, reduced glutathione levels **(F,M)**, glutathione redox status (GSH/tGSH; **G,N**), in leaves and roots of rye seedlings, respectively. Different uppercase letters represent significant differences between times in Beira, while lowercase letters represent significant differences between times in RioDeva. Values represent mean ± SD (*n* = 4).

Hydrogen peroxide levels are controlled by the activity of several enzymes (**Figures 5A–H**). Enzymatic activity analysis showed that APX was compromised in leaves and roots of both genotypes in response to Al stress (48 h; 5 mg L^-1^; **Figures 5A,E**). Catalytic activity of APX seems to be differentially regulated in both genotypes through an organ-specific manner, while CAT, GPX, and POX activities seem to be regulated in a genotype-specific manner. For both organs, CAT activity was higher in Beira genotype, while GPX and POX activities were strongly induced in RioDeva seedlings (**Figures 5B–H**). Changes in the ASC-GLU metabolism were both regulated by a genotype- and organ-specific manner (**Figures 6A–N**). ASC levels were higher in leaves of Beira seedlings, increasing 23% at the beginning of the stress (24 h; 5 mg L^-1^). No significant variations were found in ASC content of RioDeva leaves (**Figure 6A**). In roots, ASC levels were remarkably higher in RioDeva seedlings with no significant variations throughout the treatment. A positive correlation was mainly found between ASC content and ASC redox status, in leaves and roots of both genotypes (**Figures 6B,I**). MDHAR activity was remarkably higher in both organs of RioDeva seedlings and Al stress significantly increased the catalytic activity of this enzyme, around 12 and 85%, in leaves and roots, respectively (**Figures 6C,J**). DHAR activity was extraordinarily higher in both organs of Beira genotype, decreasing 30 and 12%, in leaves and roots, respectively, at the recovery period (**Figures 6D,K**). RioDeva leaves presented increased GR activity when compared to Beira and Al treatment resulted in a 29% decrease of GR activity in leaves of Beira seedlings (48 h; 5 mg L^-1^; **Figures 6E,L**). After initial exposure to Al (24 h; 5 mg L^-1^), GSH levels in leaves were very similar in both genotypes, decreasing until the end of the treatment (**Figure 6F**). The same behavior was observed in roots; however, in this organ GSH levels were higher in Beira genotype (**Figure 6M**). GSH levels and the GSH redox status were not related in leaves and roots of both genotypes (**Figures 6G,N**). Tocopherols content and TAC were higher in both organs of Beira genotype (**Figures 9A–D**). Response of ROS scavenging enzymes and non-enzymatic metabolites in the Al-tolerant and Al-sensitive genotypes, grown in the absence of Al generally demonstrated that Al phytotoxicity rather than development processes were responsible for changes in the antioxidant metabolism (Supplementary Figures S3, S4, and S7).

### Amino Acid Analyses

Compared to roots, glutamate levels were generally higher in leaves of both genotypes, presenting similar values throughout the assay, except in the control, where leaves of the tolerant genotype presented about fivefold less content of this amino acid (Supplementary Tables S1 and S2). In Beira leaves, Glu levels increased after the initial exposure to Al until the end of recovery period. RioDeva leaves manifested similar behavior; however, Glu levels increased 48 h after Al exposure (Supplementary Table S1). Roots of Beira seedlings presented higher values of Glu, which increased by 40% at the end of Al treatment and remained constant through the recovery period. Al exposure triggered an increase in Glu content in RioDeva roots at the beginning of treatment, which decreased to similar values found in the control (Supplementary Table S2). Unlike Glu, Cys, and Gly levels were higher in roots of both cultivars (Supplementary Tables S1 and S2). Comparing with the respective controls, Cys levels were fivefold higher in leaves of Beira at the end of the Al treatment, while RioDeva leaves presented an 83% decrease in this amino acid (Supplementary Table S1). Meanwhile in roots, excepting the control situation, Cys levels were higher in Beira seedlings, increasing sevenfold at early Al exposure, with values remaining constant until the recovery period. Cys content did not suffer significant variations in RioDeva roots (Supplementary Table S2). Gly levels increased 18% in leaves of Beira seedlings 24 h after Al treatment; however, late exposure of seedlings to this HM resulted in 39% decrease in Gly content that lasted until the end of the experiment (Supplementary Table S1). Early exposure of RioDeva seedlings to Al resulted in 66% decrease of Gly content in leaves. For the same genotype and organ Gly content also dropped 82% at the recovery period. As for roots, Beira and RioDeva seedlings exhibited an increase in Gly levels throughout the experiment (Supplementary Table S2). Serine levels increased 61% in Beira leaves 24 h after Al exposure. Afterward, the concentration of this amino acid decrease until the end of the experiment. Regarding roots, Beira seedlings did not display any significant variations until the recovery period where Ser levels increased 100%, when compared to control. Al treatment resulted in a decrease of Ser levels in RioDeva leaves, having the opposite effect on roots (Supplementary Tables S1 and S2).

### Estimating Photorespiration Activity – Gly/Ser Ratio

**FIGURE 7:**
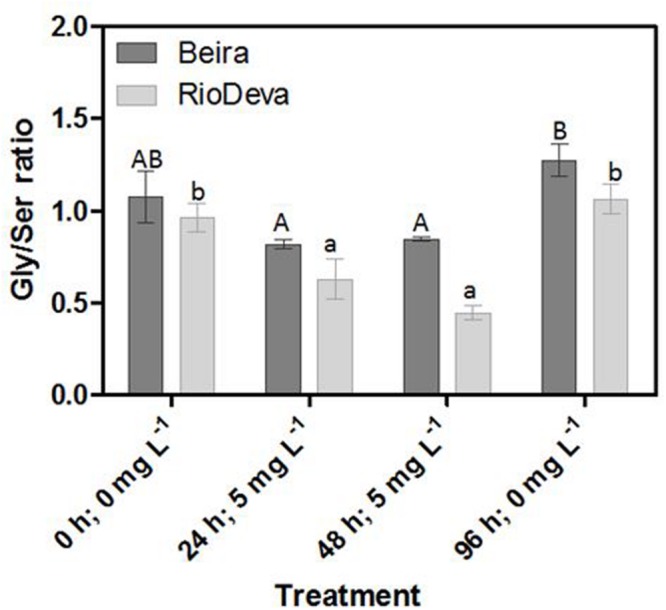
**Response of photorespiration in rye Al-tolerant (Beira) and Al-sensitive (RioDeva) genotypes.** Panel represent, the ratio of glycine and serine in leaves. Different uppercase letters represent significant differences between times in Beira, while lowercase letters represent significant differences between times in RioDeva. Values represent mean ± SD (*n* = 4).

The Gly/Ser ratio, used to estimate the level of photorespiration, decreased by 24 and 45% in leaves of Beira and RioDeva seedlings, respectively (**Figure 7**). In leaves of both genotypes Ser/Gly ratio values returned to basal levels in the recovery period. Except for the control situation, roots of Beira seedlings presented higher Gly/Ser ratio, which increased by 66%, 24 h after Al exposure until the end of the experiment (**Figure 7**). The Gly/Ser ratio was similar in seedlings grown in the absence of Al emphasizing the negative effects of this metal in both rye genotypes under short term exposure (Supplementary Figure S5).

### Oxalate Levels

**FIGURE 8:**
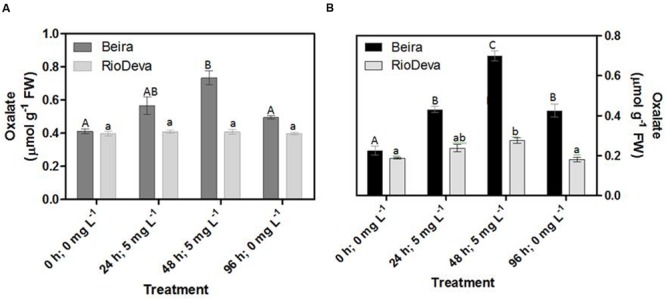
**Oxalate content in rye Al-tolerant and Al-sensitive genotypes.** Panels represent organic acid levels **(A,B)** in leaves and roots, respectively. Different uppercase letters represent significant differences between times in Beira, while lowercase letters represent significant differences between times in RioDeva. Values represent mean ± SD (*n* = 4).

After 48 h of exposure to Al, oxalate levels were 1.8- and 3- fold higher in leaves and roots of Beira seedlings, respectively (**Figures 8A,B**). No significant fluctuations were found in oxalate levels of RioDeva seedlings (**Figure 8A**). Regarding RioDeva roots, this OA increased 47% in late Al exposure, and returned to similar values found in control seedlings after the recovery period (**Figure 8B**). Seedlings grown in the absence of Al showed differences in the oxalate levels, suggesting that Al effects in the synthesis of this OA maybe potential masked by developmental effects (Supplementary Figure S6).

**FIGURE 9:**
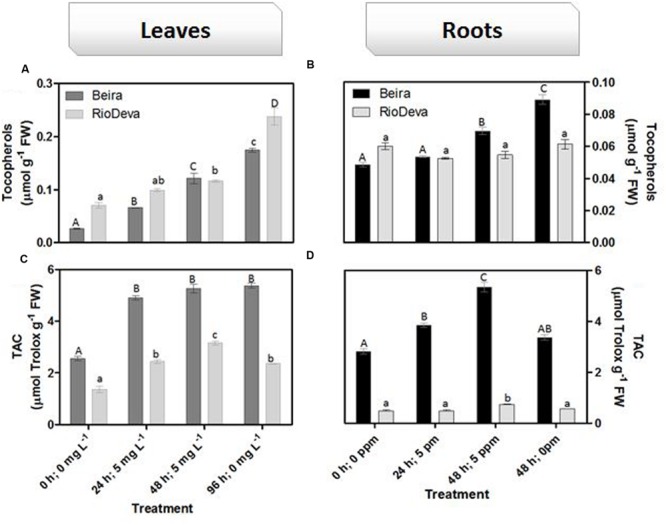
**Non-enzymatic antioxidant responses of rye Al-tolerant (Beira) and Al-sensitive (RioDeva) genotypes.** Panels represent, total tocopherols **(A,C)** and TAC **(B,D)** the in leaves and roots, respectively. Different uppercase letters represent significant differences between times in Beira, while lowercase letters represent significant differences between times in RioDeva. Values represent mean ± SD (*n* = 4).

## Discussion

Beyond affecting global climate, acid rain changes soil chemistry leading to a major accumulation of the highly phytotoxic Al^3+^ ion that compromises crop growth and yield (Poschenrieder et al., 2008; Reis et al., 2012). Measurements of Al concentration in soil solution is extremely complex and influenced by many factors. Nonetheless, Al concentrations between 2 and 5 mg L^-1^ are often found in soil solution. So, in order to simulate field conditions closely as possible, we submitted rye seedlings to a realistic 5 mg L^-1^ concentration of Al^3+^, which is already toxic to sensitive plant species (Blamey et al., 2015). To uncover the mechanisms underlying rye oxidative metabolism after Al exposure we analyzed its impact on productivity, physiology and biochemical pathways.

### Effects of Al Toxicity on Seedling Growth and Development

Al treatment affected biomass production in roots and leaves of RioDeva seedlings. Also, after the recovery period total root length was significantly reduced in this genotype. Together, these results are in agreement with the data obtained in the aluminum tolerance screening assay corroborating the previous classification of RioDeva cultivar as an Al-sensitive genotype (Gallego and Benito, 1997). Unlike RioDeva, Beira genotype seemed to overcome Al toxicity under short-term exposure, since biomass increased throughout the treatment. Considering that all the seedlings tested to Al tolerance presented root regrowth, Beira genotype was for the first time classified as Al-tolerant. Cell division and expansion are well-known processes inherent to plant growth and development. Recent studies performed in maize, confirmed that Al-induced inhibition of root growth can be due to a reduction in cell division, shortly after Al exposure (Doncheva et al., 2005). However, it is mostly believed that Al restrains cell elongation during the initial stages of root growth inhibition (Matsumoto, 2000; Horst et al., 2010). Al influences directly cell growth by binding itself to pectin molecules and DNA as well as indirectly by manipulating biochemical pathways that influence cell division and expansion. Our results predict that expansion of already divided cells plays a central role in root elongation of rye seedlings, although more detailed research is required to support this hypothesis. Cell growth is directly or indirectly affected by ascorbate and its oxidation products (MDA; DHA), as well by the enzymatic activity of peroxidases like APX (Davey et al., 2000).

In roots of Beira seedlings, higher APX activity contributes to reduce the availability of H_2_O_2_, preventing lignification of cell walls, resulting in looser cell walls (Yamamoto et al., 2003). DHA levels were higher in this genotype (data not shown) stimulating cell expansion since this radical prevents cross linking of structural proteins with hemicelluloses and polygalacturonases (Smirnoff, 1996). Additionality, some of the oxalate quantified in roots of Beira, may have been incorporated in calcium oxalate crystals, increasing cell wall plasticity (Smirnoff, 1996). ASC is related to cell division in plants. Reports showed that ASC controls transition from G1 to S phase, enhancing cell division and therefore root growth (Kerk and Feldman, 1995; Potters et al., 2000). Our results, also suggest the involvement of ASC in cell cycle progression in rye roots. Through the *Eriochrome cyanine R* staining test we observed that Al accumulated in the root apex of RioDeva roots caused irreversible damage to the root apical meristematic and cap cells, making impossible any kind of root regrowth after the recovery period. Also, due to this higher accumulation in roots of RioDeva genotype, Al can bind itself to the pectin matrix resulting in cell wall strengthening. These results are consistent with other reports of Al-induced root growth inhibition (Van et al., 1994; Tabuchi and Matsumoto, 2001; Ma et al., 2004; Eticha et al., 2005; Yang et al., 2008).

### Al and Cell Redox Homeostasis

Heavy metals (HMs) induce oxidative stress either by triggering H_2_O_2_ and ROS formation or by decreasing enzymatic and non-enzymatic antioxidants (Sharma et al., 2012). LP is considered a biomarker of metal-induced oxidative stress and lipid peroxy radicals are formed either through enzymatic processes (LOX) and/or by non-enzymatic (ROS) oxidation of membrane lipids, resulting in the major accumulation of MDA (Foyer and Noctor, 2005; Sharma et al., 2012). Lipid oxidation of biological membranes can lead to leakage of cellular components and therefore, EL assays are also commonly used to estimate membrane stability under stressful situations. LP, detected through MDA content, increased in both organs of RioDeva seedlings after Al exposure. Giannakoula et al. (2008) found similar results in maize, since Al treatment enhanced oxidation of membrane lipids in the sensitive line, while the tolerant one did not exhibited significant variations on MDA levels. Also, rice, triticale and wheat increased MDA contents after Al exposure emphasizing LP as a signal of Al toxicity in cereals (Hossain et al., 2005; Sharma and Dubey, 2007; Liu et al., 2008).

Changes in EL and LOX activity were found to be positively correlated with MDA accumulation in both organs of Beira and RioDeva genotypes, suggesting that LOX contributed in a large-scale to the formation of lipid peroxy radicals. Some reports showed that Al can change membrane lipid architecture leading to modifications in membrane permeability. It is also known that changes in membrane permeability are dependent of the plant tolerance to Al (Levine et al., 1994; Stab and Horst, 1995; Kochian and Jones, 1997; Willekens, 1997). Our results suggest that biological membranes are one of the targets of oxidative stress in rye under Al short-term exposure. As described, RioDeva seedlings accumulated more Al in its root apex than Beira’s, and this could be the major cause for the enhanced LP and EL observed in this genotype. A close relationship between LP and inhibition of the root elongation rate was already observed in soybean (Cakmak and Horst, 1991). Since Al is a non-redox metal, it cannot catalyze redox reactions inherent to the LP process. Therefore, it has been demonstrated that Al-induced rigidity of membranes facilitates the initiation of LP by the binding of iron (Fe^2+^) to membrane lipids (Oteiza, 1994; Ikegawa et al., 2000). Moreover, Al exposure can activate LOXs in plant root cells (Wang and Yang, 2005), which can explain the enhanced LP observed in RioDeva roots. Al exposure results in the impairment of antioxidant systems resulting in ROS accumulation, such as H_2_O_2_, which can culminate in the oxidation of membrane lipids (Yamamoto et al., 2002; Achary et al., 2008; Ma et al., 2012; Xu et al., 2012). These data support our results since H_2_O_2_ levels were higher in roots of RioDeva when compared to the tolerant genotype. Both genotypes presented higher H_2_O_2_ accumulation in leaves when compared to roots, which it is not surprising since due to photosynthesis electron transfer reactions are constantly leading to ROS formation (Sharma et al., 2012). After Al being removed in the recovery period, H_2_O_2_ levels decreased significantly in leaves and roots of both genotypes. This strongly suggests that H_2_O_2_ production results from direct exposure of rye seedlings to Al under short-term exposure. Undoubtedly, our results showed that the reduced H_2_O_2_ scavenging activity in both organs of RioDeva seedlings favored H_2_O_2_ accumulation leading to an imbalance on the redox homeostasis resulting in the establishment of oxidative stress in this genotype.

In order to protect themselves against oxidative damage, plants developed a powerful and complex antioxidant network comprising of both enzymatic and non-enzymatic constituents (Mittler, 2002; Foyer and Noctor, 2005; Gill and Tuteja, 2010). SOD, catalase, and APX represent the major ROS-scavenging enzymes controlling the basal levels of anion superoxide radicals (O_2_^∙-^) and H_2_O_2_ (Bowler et al., 1992; Sharma et al., 2012). H_2_O_2_ is produced by plants under normal, non-stressful conditions, through several metabolic processes playing a key role as a signaling molecule in several physiological processes and resistance tolerance (Quan et al., 2008). Enzymatic assays in roots of both genotypes showed that APX does not seems to play a central role in H_2_O_2_ scavenging of rye seedlings after Al exposure, because despite of its higher activity in RioDeva roots, no significant fluctuations were observed in its catalytic activity that could explain the significant changes in the H_2_O_2_ levels observed in this organ and genotype. This hypothesis was reinforced by the APX activity observed in leaves. Our results also demonstrated that late Al exposure (48 mg L^-1^) decreased APX activity in both organs of Beira genotype. Here, Beira seedlings can compensate APX loss with an increase in CAT activity. Since CAT has a very fast turnover rate, but a much lower affinity for H_2_O_2_ than APX it is generally accepted that this enzyme is involved in removal of H_2_O_2_ overproduced during oxidative stress, while APX is responsible for the fine modulation of H_2_O_2_ involved in signalization pathways (Bowler et al., 1992; Willekens, 1997; Mittler, 2002). Du et al. (2010) observed that in roots of soybean APX activity increased proportionality with Al concentration and treatment duration. Al-tolerant wheat genotype also presented higher APX activity after short time exposure (Xu et al., 2012) and rice increased APX activity under long term exposure (Sharma and Dubey, 2007). The same behavior was posteriorly found in rice plants 3 weeks after Al exposure (Silva et al., 2013). Al tolerance mechanisms are different between plant species, organs and tissues, which constitutes a perfectly reasonable explanation for the variation observed between the studies mentioned above (Boscolo et al., 2003).

Opposite to CAT, GPX, and POX played major roles in the first line of H_2_O_2_ detoxification in both leaves and roots of RioDeva seedlings. Under short term exposure, Al is also responsible for increased GPX and POX activities in other cereals such barley, wheat and rice (Meriga et al., 2004; Šimonovičová et al., 2004; Hossain et al., 2005). Our data demonstrate that peroxidases act as alternative and much more efficient enzymes in H_2_O_2_ detoxification then CAT in the RioDeva genotype. Also, duration of Al treatment seems to influence the response of mechanisms involved in plant protection against oxidative stress. Photorespiration is recently considered an imperative process in abiotic stress responses, since it can modulate the levels of ROS and H_2_O_2_ production (Voss et al., 2013). The glycine:serine ratio, used to estimate photorespiration activity (Kebeish et al., 2007) was lower in the RioDeva genotype, predicting a lower H_2_O_2_ production in its leaves, which does not reflect our H_2_O_2_ quantifications. This situation is easily understandable by the fact that H_2_O_2_ is produced not only in peroxisomes during photorespiration, but also in chloroplasts, cytoplasm and mitochondrias through electron transport chain reactions. H_2_O_2_ is also produced in plasma membranes by NADPH oxidase activity and in the extracellular matrix, by pH-dependent cell wall peroxidases, germins, germin-like oxlate oxidases, and amine oxidases (Slesak et al., 2007). Reduced membrane damage in Beira roots could also be due to the accumulation of the lipophilic tocopherols which are membrane-bound antioxidants. In leaves, tocopherol levels were higher in the RioDeva seedlings, suggesting that this genotype tried to overcome Al toxicity in leaves. Consistent with this results an increased antioxidant capacity in leaves and roots of the tolerant Beira genotype was observed.

### Can MDHAR and DHAR Activities Be the Key of Differential Al Tolerance in Rye Genotypes?

In plants, the ASC-GSH is crucial for the control of ROS levels and cellular redox homeostasis (Dubey, 2011). APX activity was higher in RioDeva roots as well as the ascorbate levels. Ascorbate is a potent antioxidant present in the apoplast of cells, protecting then from H_2_O_2_ and ROS generated during oxidative stress (Yin et al., 2010). Al accumulation was higher in roots of RioDeva seedlings, and since 30–90% of the total Al uptake by roots is found mainly in the apoplast of peripheral cell roots (Liu et al., 2008), it was noticed that this sensitive genotype tries to overcome Al toxicity under short term exposure by increasing total ascorbate content, in an effort to adapt to the stress situation. MDHAR specific activity was much higher than DHAR after Al exposure, suggesting that in this genotype, the MDHA radicals produced by APX in H_2_O_2_ reduction to water, were immediately transferred back into ASC via enzymatic (MDHAR) or non-enzymatic (spontaneous disproportion) processes. Reinforce this hypothesis; we observed that GR increased through the Al treatment, providing reduced gluthatione which served as an electron donor to the DHAR to reduced dehydroascorbate back into ASC, because even when a fast disproportion of the MDHA radical occurs some DHA is always produced.

Together these results suggest that the Al-sensitive RioDeva genotype tries to counteract the negative effects of Al toxicity in roots by increasing the ASC content, although without success because oxidative stress biomarkers and biomass were deeply affected by Al exposure. Beira roots seemed to cope with Al toxicity by another more effective strategy. In this genotype, despite of its higher catalytic activity when compared to MDHAR, DHAR enzyme did not exhibit significant variations after the Al treatment. This fact, combined with the poor ASC regeneration and lower GR activity, suggests that DHA generated through APX activity is being converted into oxalate and tartarate, lowering Al concentration in root tissues by the formation of stable and non-toxic Al-oxalate conjugates that are posteriorly translocated into the vacuoles. Supporting this hypothesis is the enhanced accumulation of oxalate in roots of this Al-tolerant genotype after Al exposure. Our results are in accordance with other reports obtained in maize (Klug et al., 2011) and buckwheat (Zhu et al., 2015 and references).

Regarding leaves the same behavior was observed with the exception that in this organ Beira genotype is also investing in the ASC regeneration, thus providing an extra protection for the photosynthetic apparatus against the Al toxicity. Another fact is that Al affected not only the GR activity in leaves and roots of both genotypes, but also the biosynthetic pathway of the amino acids that constitute GSH. We observed that serine levels were close to those of glycine, reinforcing the fact that GSH main coexist in two different tripeptides in rye (Rauser, 1999).

## Conclusion

We observed that rye Al tolerance under short term exposure is dependent on the genotype and plant organs, and that the response of the antioxidant system comprises changes in proline and ascorbate levels, its oxidation products and its regenerating enzymes, being key points for the survival of rye seedlings in early development stages in Al contaminated soils.

## Author Contributions

AS conceived the project and experiments and wrote the article with contributions of all authors; HA and AS conducted all the experiments; AH supervised the experiments and revised the manuscript; JT supervised the experiments and revised the manuscript; MM conceived the project and supervised the experiments and revised the manuscript; FF conceived the project and supervised the experiments and revised the manuscript.

## Conflict of Interest Statement

The authors declare that the research was conducted in the absence of any commercial or financial relationships that could be construed as a potential conflict of interest.

## Abbreviations

Al: aluminum
APX: ascorbate peroxidase
ASC: ascorbate
CAT: catalase
Cys: cysteine
DHAR: dehydroascorbate reductase
EL: electrolyte leakage
Glu: glutamate
Gly: glycine
GPX: guaiacol peroxidase
GR: gluthatione reductase
GSH: glutathione
H_2_O_2_: hydrogen peroxide
LOX: lipoxygenase
MDA: malondialdehyde
MDHAR: monodehydroascorbate reductase
POX: phenol peroxidase
Ser: serine
TAC: total antioxidant capacity
