# Supplementary material for: Oxidative Metabolism of Rye (Secale cereale L.) after Short Term Exposure to Aluminum: Uncovering the Glutathione–Ascorbate Redox Network
Source: Front Plant Sci. 2016 May 24;7:685. doi: 10.3389/fpls.2016.00685 (PMC4877395; doi:10.3389/fpls.2016.00685)
Supplement: Supplementary file 1 [file Data_Sheet_1.PDF]

Table S1. Amino acid analysis of glutathione´s constituent amino acids (glutamate – Glu, cysteine – Cys, glycine – Gly and serine – Ser) in leaves of Beira and RioDeva cultivars. Different uppercase letters represent significant differences between times in Beira, while lowercase letters represent significant differences between times in RioDeva. Values represent mean ± SD (n=4).

**Leaves**		Treatment			
α a.a	Variety	0 h; 0 mg L^{-1}	24 h; 5 mg L^{-1}	48 h; 5 mg L^{-1}	96 h; 0 mg L^{-1}
Glu	B	1.91 ± 0.12^{A}	6.17 ± 0.06^{B}	13.42 ± 2.09^{C}	19.96 ± 0.70^{D}
	Rd	9.44 ± 0.52^{a}	7.43 ± 1.01^{a}	13.00 ± 0.55^{b}	27.67 ± 2.63^{c}
Cys	B	0.01 ± 0.004^{A}	0.02 ± 0.01^{A}	0.05 ± 0.002^{B}	0.02 ± 0.008^{A}
	Rd	0.06 ± 0.02^{b}	0.03 ± 0.01^{ab}	0.01 ± 0.001^{a}	0.01 ± 0.004^{a}
Gly	B	0.68 ± 0.07^{A}	0.80 ± 0.12^{B}	0.49 ± 0.04^{A}	0.53 ± 0.08^{A}
	Rd	1.30 ± 0.07^{c}	0.44 ± 0.06^{b}	0.48 ± 0.06^{b}	0.44 ± 0.02^{b}
Ser	B	0.62 ± 0.05^{B}	1.00 ± 0.06^{C}	0.57 ± 0.03^{B}	0.37 ± 0.05^{A}
	Rd	1.37 ± 0.13^{b}	0.79 ± 0.07^{a}	0.47 ± 0.07^{a}	0.54 ± 0.10^{a}

Table S2. Amino acid analysis of glutathione´s constituent amino acids (glutamate – Glu, cysteine – Cys, glycine – Gly and serine – Ser) in roots of Beira and RioDeva cultivars. Different uppercase letters represent significant differences between times in Beira, while lowercase letters represent significant differences between times in RioDeva. Values represent mean ± SD (n=4).

**Roots**		Treatment			
α a.a	Variety	0 h; 0 mg L^{-1}	24 h; 5 mg L^{-1}	48 h; 5 mg L^{-1}	96 h; 0 mg L^{-1}
Glu	B	5.13 ± 0.37[A]	5.15 ± 0.12[A]	7.13 ± 0.62[B]	6.66 ± 0.83[B]
	Rd	3.85 ± 0.52[a]	5.52 ± 0.3[b]	4.74 ± 0.50[a]	5.16 ± 0.41[a]
Cys	B	0.003 ± 0.0003[A]	0.02 ± 0.005[C]	0.02 ± 0.002[BC]	0.01 ± 0.002[AB]
	Rd	0.008 ± 0.001[a]	0.005 ± 0.002[a]	0.004 ± 0.001[a]	0.009 ± 0.002[a]
Gly	B	0.46 ± 0.07[A]	0.91 ± 0.10[B]	0.86 ± 0.08[B]	2.88 ± 0.83[C]
	Rd	0.76 ± 0.17[a]	0.97 ± 0.03[b]	0.94 ± 0.04[b]	0.98 ± 0.04[b]
Ser	B	0.49 ± 0.06[A]	0.42 ± 0.01[A]	0.48 ± 0.03[A]	0.98 ± 0.03[B]
	Rd	0.46 ± 0.11[a]	0.67 ± 0.06[b]	0.70 ± 0.07[b]	0.61 ± 0.01[b]

Fig S1

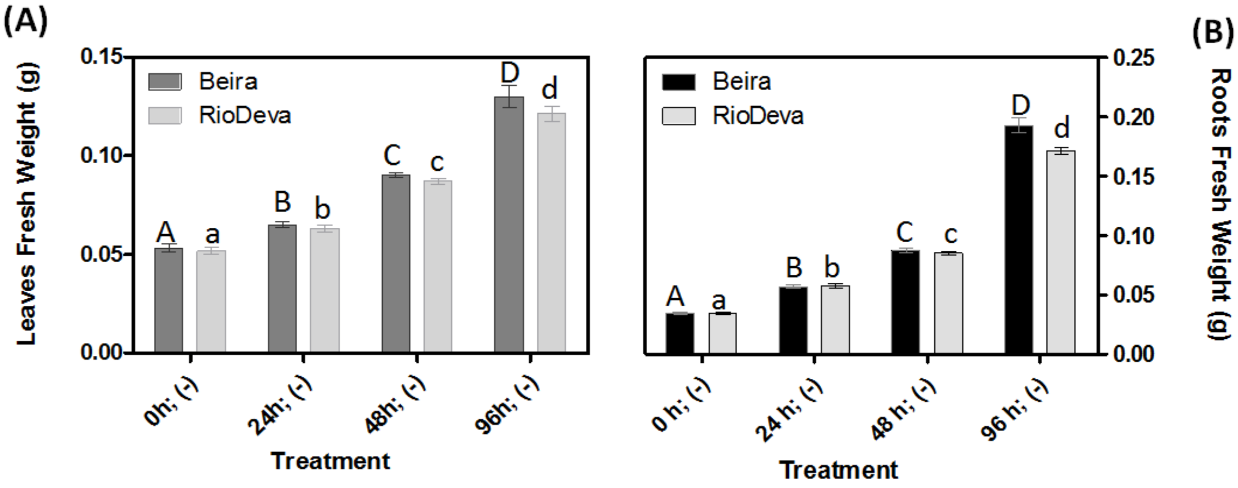

(A)
Leaves Fresh Weight (g)
0.15
0.10
0.05
0.00
Beira
RioDeva
A
a
B
b
C
c
D
d
0h; (-)
24h; (-)
48h; (-)
96h; (-)
Treatment
(B)
Roots Fresh Weight (g)
0.25
0.20
0.15
0.10
0.05
0.00
Beira
RioDeva
A
a
B
b
C
c
D
d
0 h; (-)
24 h; (-)
48 h; (-)
96 h; (-)
Treatment

Fig S2

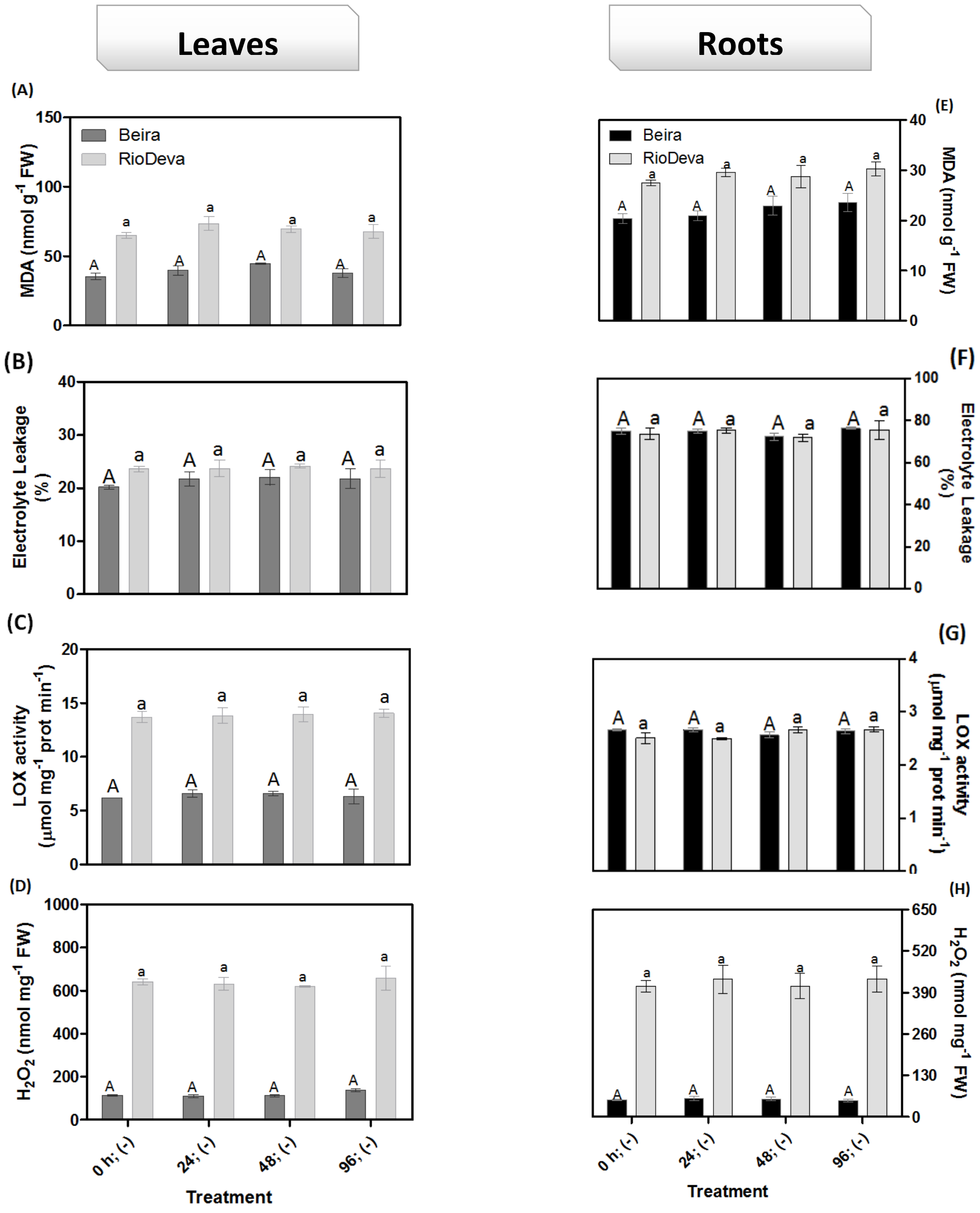
Leaves
Roots
(A)
MDA (nmol g-1 FW)
Beira
RioDeva
(B)
Electrolyte Leakage (%)
(C)
LOX activity (µmol mg-1 prot min-1)
(D)
H2O2 (nmol mg-1 FW)
(E)
(F)
(G)
(H)
0 h; (-)
24; (-)
48; (-)
96; (-)
Treatment

Fig S3

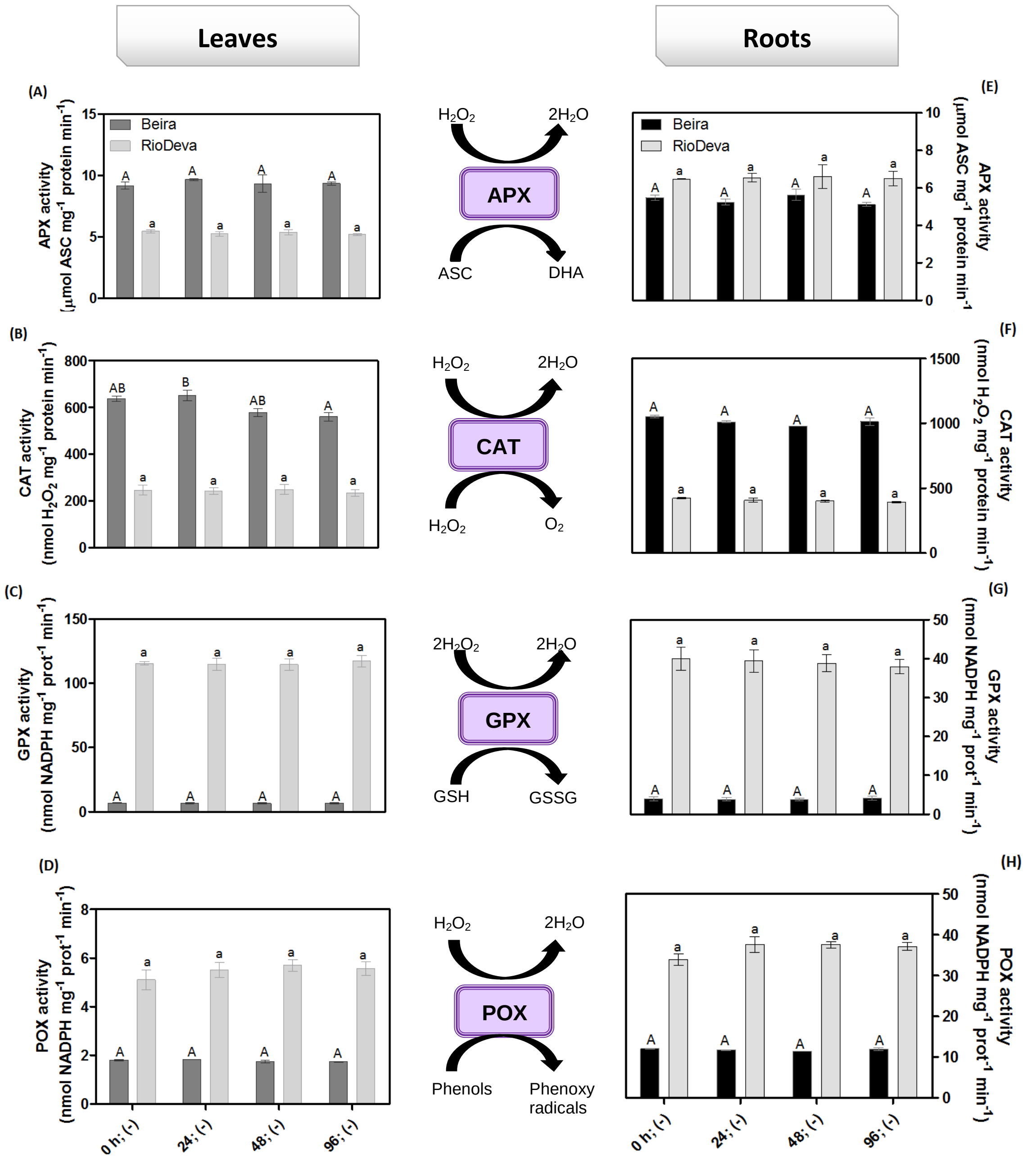
Leaves
Roots
(A)
(B)
(C)
(D)
(E)
(F)
(G)
(H)
Beira
RioDeva
APX activity (µmol ASC mg-1 protein min-1)
CAT activity (nmol H2O2 mg-1 protein min-1)
GPX activity (nmol NADPH mg-1 prot-1 min-1)
POX activity (nmol NADPH mg-1 prot-1 min-1)
H2O2
2H2O
APX
ASC
DHA
CAT
H2O2
O2
2H2O2
GPX
GSH
GSSG
POX
Phenols
Phenoxy radicals
0 h; (-)
24; (-)
48; (-)
96; (-)

Fig S4

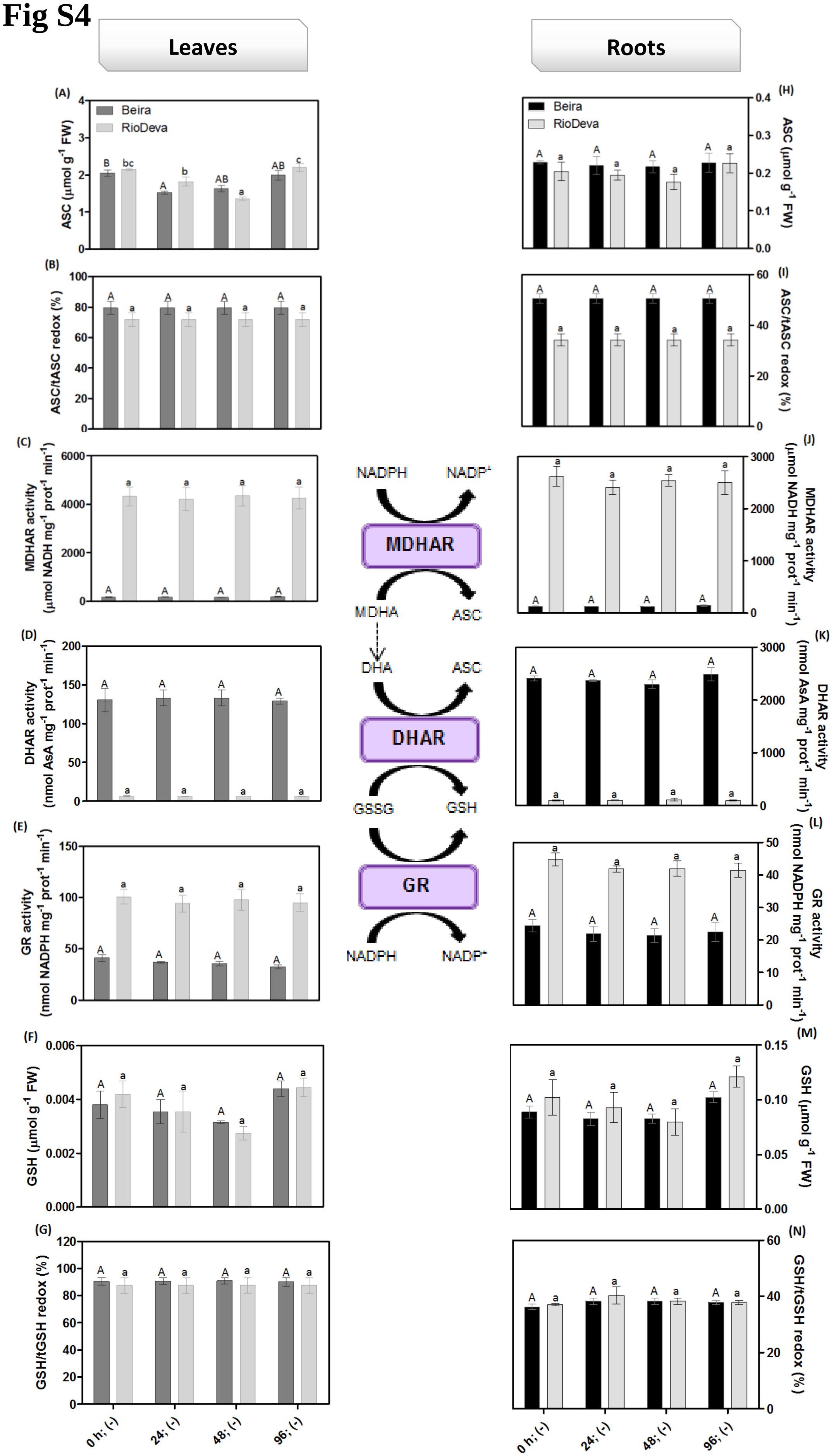
Leaves
Roots
Beira
RioDeva
ASC (µmol g-1 FW)
ASC/tASC redox (%)
MDHAR activity (µmol NADH mg-1 prot-1 min-1)
DHAR activity (nmol AsA mg-1 prot-1 min-1)
GR activity (nmol NADPH mg-1 prot-1 min-1)
GSH (µmol g-1 FW)
GSH/tGSH redox (%)
NADPH
NADP+
MDHAR
MDHA
ASC
DHA
DHAR
GSSG
GSH
GR
0 h; (-)
24; (-)
48; (-)
96; (-)

Fig S5

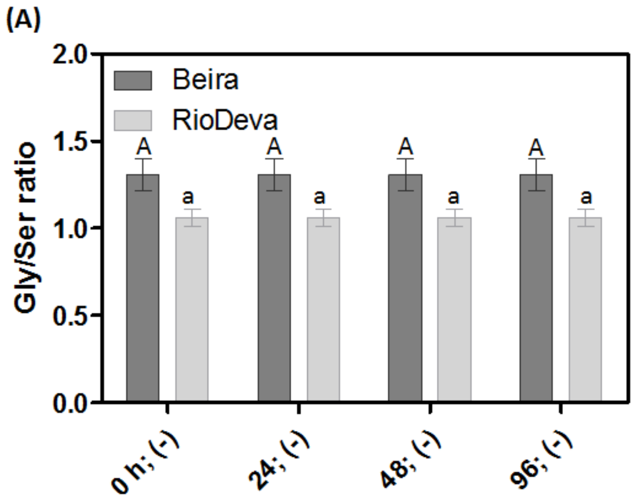

Fig S6

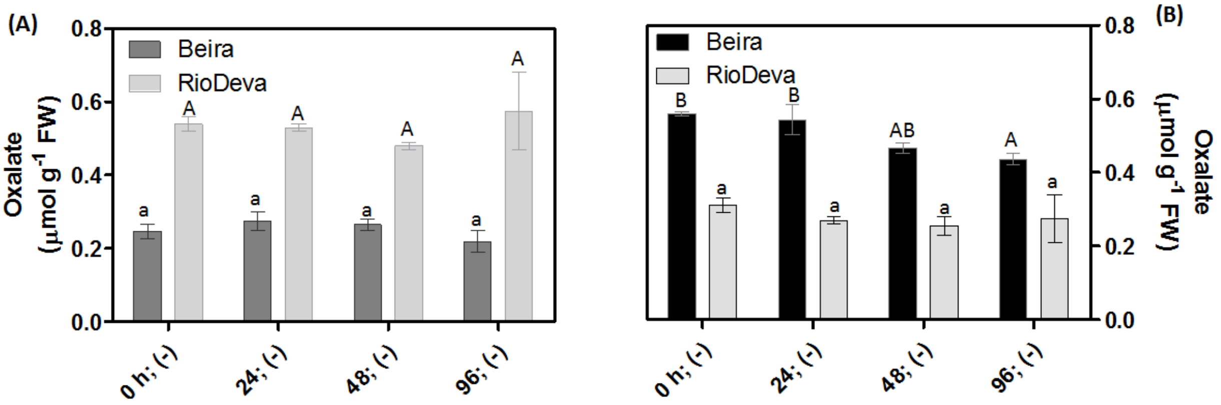
(A)
Oxalate
(µmol g-1 FW)
0.8
0.6
0.4
0.2
0.0
Beira
RioDeva
a
A
0 h; (-)
24; (-)
48; (-)
96; (-)
(B)
B
AB

Fig S7

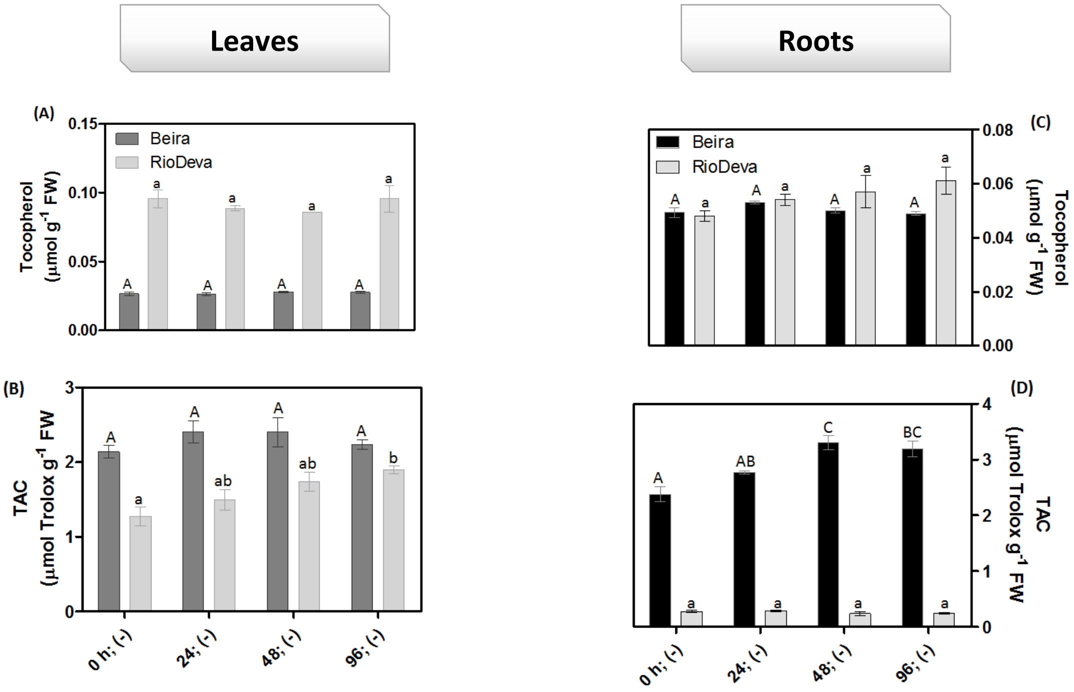

Leaves
Roots
(A)
(B)
(C)
(D)
Beira
RioDeva
Tocopherol (µmol g-1 FW)
TAC (µmol Trolox g-1 FW
0 h; (-)
24; (-)
48; (-)
96; (-)

Fig S1. Biometric analysis: fresh weight in leaves (A) and roots (B) of rye Al-tolerant (Beira) and Al-sensitive (RioDeva) genotypes, in seedlings grown without Al. Different uppercase letters represent significant differences between times in Beira, while lowercase letters represent significant differences between times in RioDeva. Values represent mean ± SD (n=30). Asterisk represents significant differences between genotypes in root length. Values represent mean ± SD (n=25).

Fig S2. Oxidative stress parameters of rye Al-tolerant (Beira) and Al-sensitive (RioDeva) genotypes. Lipid peroxidation (MDA) content (A, E), electrolyte leakage (B, F), LOX activity (C, G) and hydrogen peroxide (H_2O_2) levels in leaves and roots of rye seedlings, respectively, grown without Al. Different uppercase letters represent significant differences between times in Beira, while lowercase letters represent significant differences between times in RioDeva. Values represent mean ± SD (n=4).

Fig S3. Response of ROS scavenging enzymes in rye Al-tolerant (Beira) and Al-sensitive (RioDeva). Panels represent ascorbate peroxidase (APX) activity (A, E), catalase (CAT) activity (B, F), guaiacol peroxidase (GPX) activity (C, G) and phenol peroxidase (POX) activity (D, H), in leaves and roots of rye seedlings, respectively, grown without Al.. Different uppercase letters represent significant differences between times in Beira, while lowercase letters represent significant differences between times in RioDeva. Values represent mean ± SD (n=4).

Fig S4. Response of ROS scavenging enzymes in rye Al-tolerant (Beira) and Al-sensitive (RioDeva). Panels represent ascorbate peroxidase (APX) activity (A, E), catalase (CAT) activity (B, F), guaiacol peroxidase (GPX) activity (C, G) and phenol peroxidase (POX) activity (D, H), in leaves and roots of rye seedlings, respectively, grown without Al. Different uppercase letters represent significant differences between times in Beira, while lowercase letters represent significant differences between times in RioDeva. Values represent mean ± SD (n=4).

Fig S5. Response of photorespiration in rye Al-tolerant (Beira) and Al-sensitive (RioDeva) genotypes. Panel represent, (A) the ratio of glycine and serine in leaves of seedlings grown without Al. Different uppercase letters represent significant differences between times in Beira, while lowercase letters represent significant differences between times in RioDeva. Values represent mean ± SD (n=4).

Fig S6. Oxalate content in rye Al-tolerant and Al-sensitive genotypes. Panels represent organic acid levels (A, B) in leaves and roots, respectively, of seedlings grown without Al. Different uppercase letters represent significant differences between times in Beira, while lowercase letters represent significant differences between times in RioDeva. Values represent mean ± SD (n=4).

Fig S7. Non-enzymatic antioxidant responses of rye Al-tolerant (Beira) and Al-sensitive (RioDeva) genotypes. Panels represent, total tocopherols (A, C) and total antioxidant capacity (TAC) (B,D) the in leaves and roots, respectively, of seedlings grown without Al . Different uppercase letters represent significant differences between times in Beira, while lowercase letters represent significant differences between times in RioDeva. Values represent mean ± SD (n=4).
